# Manipulating Insect Sex Determination Pathways for Genetic Pest Management: Opportunities and Challenges

**DOI:** 10.3389/fbioe.2022.867851

**Published:** 2022-06-28

**Authors:** Alex Siddall, Tim Harvey-Samuel, Tracey Chapman, Philip T. Leftwich

**Affiliations:** ^1^ School of Biological Sciences, University of East Anglia, Norwich, United Kingdom; ^2^ Arthropod Genetics, The Pirbright Institute, Pirbright, United Kingdom

**Keywords:** gene drive, sex conversion, release of insects carrying a dominant lethal, sterile insect technique (SIT), doublesex (dsx), *tra*, dosage compensation, sex determination’

## Abstract

Sex determination pathways in insects are generally characterised by an upstream primary signal, which is highly variable across species, and that regulates the splicing of a suite of downstream but highly-conserved genes (*transformer*, *doublesex* and *fruitless*). In turn, these downstream genes then regulate the expression of sex-specific characteristics in males and females. Identification of sex determination pathways has and continues to be, a critical component of insect population suppression technologies. For example, “first-generation” transgenic technologies such as fsRIDL (Female-Specific Release of Insects carrying Dominant Lethals) enabled efficient selective removal of females from a target population as a significant improvement on the sterile insect technique (SIT). Second-generation technologies such as CRISPR/Cas9 homing gene drives and precision-guided SIT (pgSIT) have used gene editing technologies to manipulate sex determination genes *in vivo*. The development of future, third-generation control technologies, such as Y-linked drives, (female to male) sex-reversal, or X-shredding, will require additional knowledge of aspects of sexual development, including a deeper understanding of the nature of primary signals and dosage compensation. This review shows how knowledge of sex determination in target pest species is fundamental to all phases of the development of control technologies.

## Introduction

Insect pests cause enormous damage to human health (through the transmission of diseases such as dengue fever and malaria) and agriculture (through damage to crops or livestock). Existing control methods include pesticides, biological control, and integrated pest and habitat management. However, while many of these approaches have been highly successful, they also have limitations. For example, the use of pesticides can select strongly for resistance, damage non-pest populations (Hawkins et al., 2018) and the environment. The success of biological control and integrated management programmes may also depend upon whether efficient natural enemies are available and the specific ecological setting. As a result, existing control strategies, particularly chemical control, are likely to become increasingly restricted while simultaneously becoming less effective. Global climate change is also predicted to increase the range and the number of insect pests (Deutsch et al., 2018; Gomez-Zavaglia et al., 2020; Sultana et al., 2020). Therefore, it is clear that there are significant challenges for the future in controlling insect pests, to safeguard against disease and maintain global food security.

In light of these concerns, there has been considerable investment in new and alternative technologies, such as genetics-based approaches to pest control, to protect health and food security (Alphey, 2014; Burt and Crisanti, 2018; Raban et al., 2020). Genetic Pest Management (GPM) aims to harness the natural mating system of the pest species to introduce into the target population traits that will reduce fitness and ultimately lead to a reduction of numbers or elimination. The most widely used GPM systems for suppression to date have been variants of the sterile insect technique (SIT) (Klassen, 2005), including the Wolbachia incompatible insect technique (IIT) (Atyame et al., 2011; De Barro et al., 2011; Zheng et al., 2019) and genetic engineering (Phuc et al., 2007). GPM systems that are transmitted or inherited through one sex and sterilise, kill or change the sex of the other offer the most significant potential for control (Bax and Thresher, 2009). As females are predominantly the agents of damage (*via* biting or ovipositing), and generally determine the effective population size, most approaches have focused on releasing benign males that produce either male progeny or none at all (Rendón et al., 2004). Elimination of females ensures long-term suppression and immediately reduces the associated damage caused by biting or egg-laying.

GPM technologies for insect population suppression currently under development seek to improve on older systems by spreading female-targeting genetic loads through a population or converting female progeny into functional males. These newer technologies also make wide use of contemporary molecular biology tools—particularly those involved in gene editing such as CRISPR/Cas9. However, what is common to all is that they exploit knowledge of the sex determination pathways of the target species, thus exemplifying the importance of incorporating fundamental biological principles to underpin applied science in GPM (Leftwich et al., 2016; Leftwich et al., 2021).

Here we first introduce the fundamentals of insect sex determination systems, focusing on species of interest to GPM. We detail which components are conserved and which show more rapid evolution, what types of primary signals have evolved and in which “direction” they push downstream cascades (e.g., towards maleness or femaleness). We then provide a framework for understanding how sex determination systems have been used to develop insect population suppression tools. We describe three “generations” of genetic engineering technologies with related components or goals. First-generation systems are genetically engineered analogues of the classical Sterile Insect Technique. Second-generation systems are under development and are made possible by the development of the CRISPR/Cas9 platform. Finally, we discuss the challenges inherent in developing “third-generation” control technologies that seek to achieve the goal of sex-conversion by manipulating master regulators of sex determination.

## Insect Sex Determination Systems

**FIGURE 1:**
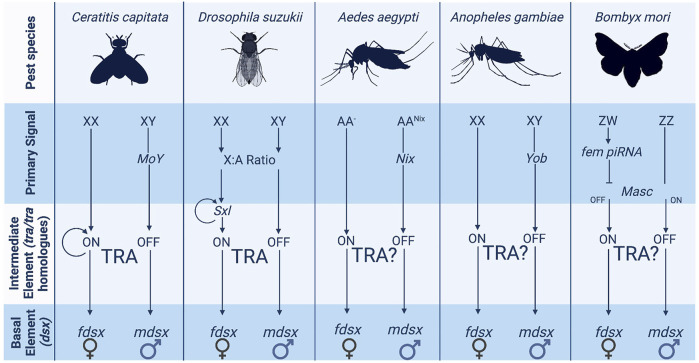
The variety of sex determination systems employed by pest insects. The more upstream elements of the sex determination pathway such as the primary signal vary widely across pest insect species. Several species have XX/XY chromosome structures although they do not utilise them in the same way. Ceratitis and Aedes employ masculinizing elements carried on the Y chromosome whereas Drosophila uses X counting to determine sex.

Sex determination systems can be described as a cascade in the form of a pyramid. There is an initial primary signal (“master regulator”—top of the pyramid) that initiates a limited series of intermediary elements (middle) that then result in diverse downstream sexual differentiation and development (base of the pyramid). In insects, the genes at the base of the pyramid tend to be highly conserved, while the elements at the top show marked diversity, both in identity and function (Adolfi et al., 2021; Hopkins and Kopp, 2021). The hypothesis is that these basal genes—which generally consist of transcription factors—represent ancient mechanisms of sex determination [e.g., *doublesex* is shared with some non-dipteran arthropods (Price et al., 2015; Wexler et al., 2019)]. At the same time, the primary signal can evolve rapidly, even within a taxonomic order. The differences in conservation between the basal and intermediate elements of the sex determination pathway are shown in Figure 1.

**FIGURE 2:**
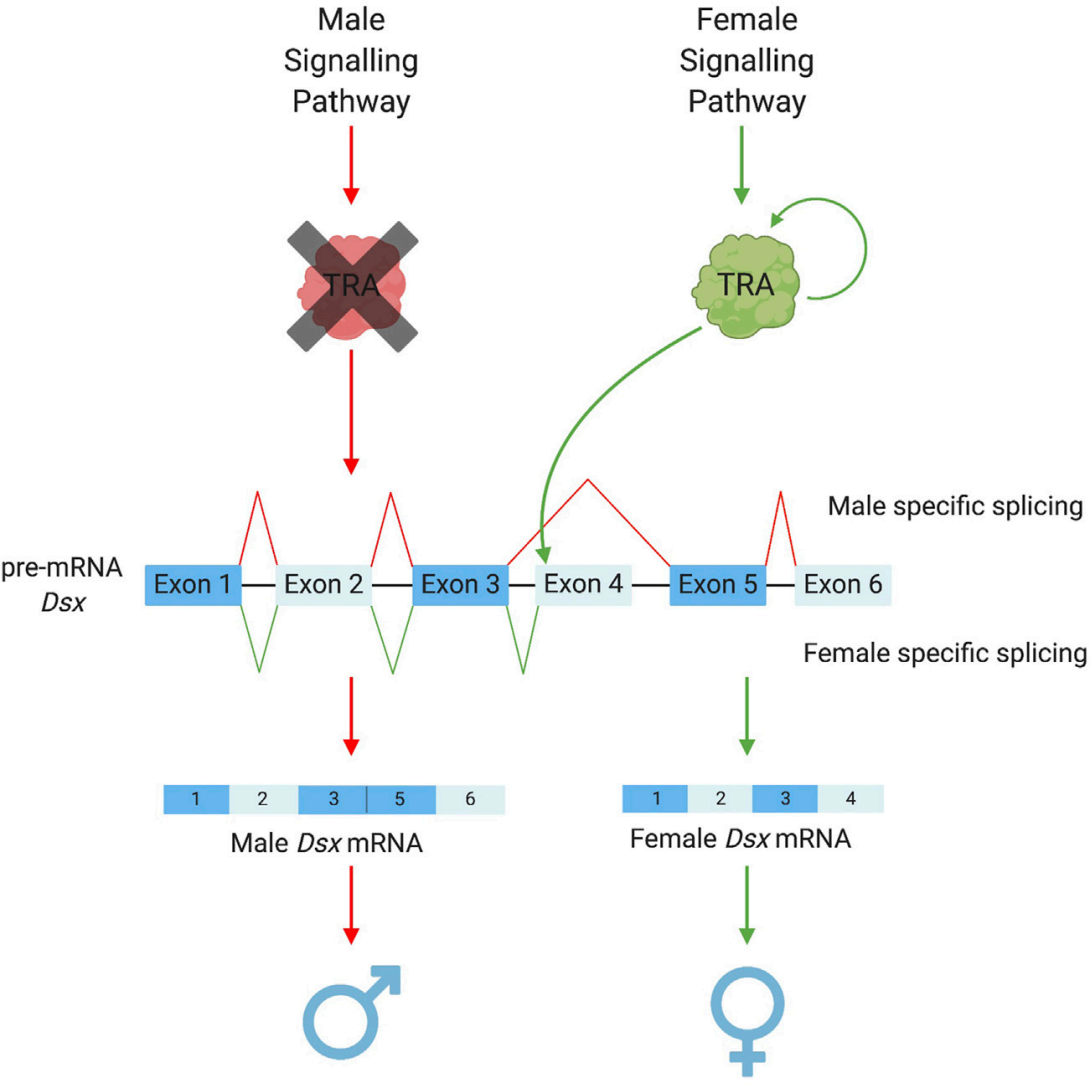
Sex-specific splicing patterns of doublesex. The conserved elements of the sex determination pathway that result in the alternative splicing of the pre-mRNA dsx. Depending on the upstream signalling pathways TRA is either present as a functional protein (in female development) or a non-functional protein (in male development). TRA acts as a splicing enhancer to promote the recruitment of the splicing machinery to the weak splice acceptor prior to exon 4 of the dsx pre-mRNA (Shukla and Nagaraju, 2010). This allows for the retention of exon 4 in the mRNA resulting in the female version of dsx. Doublesex exon numbers vary among species with the use of splicing to retain or remove sex-specific exons remaining constant, Ceratitis capitata pathway illustrated in this figure.

Amongst the downstream basal elements, the most well conserved is *doublesex (dsx)* (Saccone et al., 2002; Price et al., 2015; Verhulst and van de Zande, 2015; Wexler et al., 2019)*.* Ubiquitous amongst insects, *dsx* is the “central nexus” between sex determination and sexual differentiation cascades (Verhulst and van de Zande, 2015). It functions as a transcription factor activating or repressing thousands of downstream genes which cause female or male somatic differentiation. Its role in this regulation (male or female biasing) is determined by whether it exists in a male or female “form” as a protein. In most cases, this is determined by sex-specific alternative splicing of the initial *dsx* pre-mRNA—itself determined by intermediary regulators between the primary signal and *dsx*. “Male” *dsx* typically represents the constitutive splicing isoform; while female-specific *dsx* isoforms require the splice enhancing factor transformer (*tra*) (Figure 2). However, even within this most conserved member of the insect sex determination cascade, some variation does exist. For example, there are significant differences in the number and “style” of exon skipping between different insect species (Verhulst and van de Zande, 2015). For example, in lepidopterans, the constitutive dsx isoform is female with male determining factors required to shift splicing towards a male form (Lee et al., 2015; Xu et al., 2017; Visser et al., 2021). Further, in at least two species of termites, *dsx* has evolved towards male-only expression rather than sex-alternate splicing (Wexler et al., 2019; Miyazaki et al., 2021).

An intermediary element that exists directly above *dsx* is *transformer* (*tra*). Although not as highly conserved as *dsx* (Verhulst et al., 2010b)*, tra* homologues have been identified in a variety of insect orders, e.g., Coleopterans [*Tribolium castaneum* (Shukla and Palli, 2012)], Hymenopterans [*Apis mellifera* (Gempe et al., 2009), *Nasonia vitripennis* (Verhulst et al., 2010a)] and dipterans [*Drosophila melanogaster* (Sosnowski et al., 1989; Inoue et al., 1990), *Musca domestica* (Inoue and Hiroyoshi, 1986) and a number of Tephritid fruitflies (Pane et al., 2002; Lagos et al., 2007; Ruiz et al., 2007)]. In these groups, sex-specific splicing of *tra* leads to a “functional” female-specific or “non-functional” male-specific protein. Interestingly, *tra* has often been found to self-regulate its splicing (except in *D. melanogaster*) acting as a positive self-regulatory element to increase its own expression (Pane et al., 2002; Gempe et al., 2009; Salvemini et al., 2009; Verhulst et al., 2010a; Hediger et al., 2010). Functional *tra* acts as a splicing enhancer, binding *dsx* pre-mRNA and promoting the inclusion of female-specific exons in the final transcript.

Although *tra* is an essential gene in the sex determination pathway of many dipterans and hymenopterans; in other species, there may be, as yet, no identified homologue, as is the case for *Aedes aegypti* (Nene et al., 2007). In these cases, there may be functional quasi-equivalents for *transformer*. For example, in silkmoth (*Bombyx mori*) males, P-element somatic inhibitor and IGF-II mRNA binding proteins interact to form a complex which binds *dsx* pre-mRNA. This complex inhibits internal splice site junctions, excluding female-specific exons to produce “male” form dsx mRNA (Suzuki et al., 2010). This example illustrates that while there may be orthogonal splicing factor/s, analogous to *tra*, the role the new factor/s plays may be very different (promoting male-form, rather than female-form *dsx* alternate splicing). The *transformer-2* gene (*tra2*) is also involved in the sex determination pathway of many insects. It is often an additional factor that forms an essential part of the splicing enhancer complex, which helps sustain and regulate the splicing of *tra* (Salvemini et al., 2009). It is, however, not a homologue of *transformer* itself. *Tra-2*, unlike *tra,* has also been shown to have both expression and function in males (Salvemini et al., 2009)*.*

Above *tra* (or other intermediary elements) in the sex determination pathway lies the primary signal or master regulator underpinning the sexual determination cascade. The identity and function of these master regulators vary enormously between species even within the same order due to a high turnover rate of the primary signaler at this level (Gempe and Beye, 2011). For example, in four dipteran species, the mosquitos *Anopheles gambiae*, *Ae. aegypti,* the Mediterranean fruitfly *Ceratitis capitata*, and the house fly *M. domestica*, the master regulators of sex determination are evolutionarily unrelated [*Yob, Nix, MoY*, and *Mdmd* respectively (Hall et al., 2015; Krzywinska et al., 2016; Sharma et al., 2017; Meccariello et al., 2019)]. While the exact mechanisms by which these primary signals act remains largely unknown, the mosquito species assessed so far (including those listed above) do not appear to possess a *tra* homologue (Nene et al., 2007). In contrast, the sex-specific splicing of *tra* is integral to the sex determination cascade in *C. capitata and M. domestica*, strongly suggesting divergent functions in regulating intermediary elements between the top and bottom levels of the pyramid in mosquitos and other diptera (Saccone et al., 2011). In *D. melanogaster*, sex is determined by an X-chromosome counting mechanism. The expression ratios of specific X-linked (*sis-a, sis-b, sis-c* and *run*) and autosomal genes determine the correct expression of an autoregulatory-splicing female-determining gene (*sex-lethal*) (Cline, 1993; Kaiser and Bachtrog, 2010). A 1:1 X: A ratio (implying two X chromosomes) leads to functional sex-lethal expression and the female sex determination cascade (Baker and Ridge, 1980; Sánchez and Nöthiger, 1982; Parkhurst et al., 1990). Although *tra* plays a crucial intermediary role in *D. melanogaster* (as in *C. capitata*), the sex-determining role of *sex-lethal* appears to be Drosophilid-specific (Meise et al., 1998). In Lepidoptera, primary signals can vary, as both Z-chromosome counting and dominant primary signals exist in different species (Traut et al., 2007). In *B. mori*, a dominant female-linked (*feminizer*) piRNA system encoded on the female-specific W chromosome silences Z-linked genes that would otherwise initial male sex-determination (Hasselmann et al., 2008; Katsuma et al., 2018); this then directs *dsx* splicing. In Hymenoptera, sexual fate is effectively regulated by the presence or absence of a paternal genome. E.g., in the honeybee, *A. mellifera*, it is determined by heterozygosity at a single locus the complementary sex determiner (csd) gene (Gempe et al., 2009), single sex alleles within an organism result in male development (homozygous/hemizygous) and mismatched sex alleles develop into females (heterozygous). While in the haplodiploid wasp *N. vitripennis* the sex determination gene *wasp overruler of masculinization* (*wom*) is only transcribed from the paternally provided genome (Zou et al., 2020). In both systems, *tra* and *dsx* are still employed as intermediate and basal elements (Cho et al., 2007; Zou et al., 2020).

The genomic location of the master regulator and, specifically, whether it exists on a heteromorphic sex chromosome is a further important aspect of insect sex determination systems in this review. In many cases, the evolution of distinct sex-chromosomes necessitates a mechanism for equalising the expression of shared, sex-linked genes between sexes (dosage compensation). In *D. melanogaster*, the absence of *sex-lethal* in males initiates hypertranscription from the single X-chromosome to make up for two X’s in females. Inactivation of *sex-lethal* in females leads to deadly X-chromosome hypertranscription due to carrying two X chromosomes. This “coupled” sex determination and dosage compensation has implications for manipulating these systems for genetic pest control. For example, it is a significant challenge to aim to alter sexual fate without simultaneously “programming” the dosage compensation pathway. As a result, in many cases where the master regulator has been identified and ectopically expressed in females (e.g., *Yob* in *An. gambiae; Guy1 in Anopheles stephensi*), the result is the death of the XX individuals rather than their conversion to fertile males (Criscione et al., 2016; Krzywinska and Krzywinski, 2018; Qi et al., 2019). This represents a high hurdle if the most potent application of manipulating sex determination for GPM suppression systems is to convert a population to a single-sex rather than selectively kill off females.

Fortunately, neither fully differentiated sex chromosomes, nor coupled-dosage compensation pathways where heterogametic sex chromosomes exist, appear universal amongst insects that are of concern to human health or agriculture. For example, *Aedes albopictus* (and other culicine mosquitos) do not possess heterogametic sex chromosomes but rather a small “Male-determining” locus on chromosome 1 (Hall et al., 2014; Gomulski et al., 2018). Transgenic expression of the male-determining gene from within this locus (*Nix*) in transgenic *Ae. albopictus* was sufficient to convert females into functioning males (Lutrat et al., 2022). Similarly, in *C. capitata*, transient ectopic expression of *MoY* is enough to convert karyotypic females to functional males, suggesting either a lack of dosage compensation or an “un-coupled” version in this species (Meccariello et al., 2019).

Understanding the nature of the sex determination pathway that has evolved in a pest of concern, and its possible interaction with dosage compensation, provides some potential routes for manipulating a species for genetic pest management. If the goal is female to male sex *conversion*, then the upper levels of the pyramid will likely need to be manipulated to ensure complete sex conversion and the viability/fertility of converted individuals. However, this goal may be difficult or practically impossible for species with lethal dosage compensation. If the goal is simply to kill one of the sexes, then lower, more conserved levels of the pyramid can be targeted, and this may also be beneficial in transferring efficient designs between related pests. Further exploration of the fundamental basis of sex determination mechanisms is, therefore, essential.

## First-Generation Technologies: An Improvement on the Past

First-generation transgenic GPM systems are genetically engineered analogues of the classical sterile insect technique (SIT). In the SIT, the mass release of irradiated (sterilised) males results in a lower proportion of fertilised females in the field due to mating with the sterile males instead of the fertile, wildtype males. SIT is most efficient if only males are released (Rendón et al., 2004). Preventing the introduction into the population of females that can damage fruit crops or transmit disease is an additional advantage. However, male-only releases require an efficient mechanism for sex sorting. For this purpose, genetic sexing strains (GSS- Box 1) were developed that differentiated between males and females with selective markers such as pupal colour or conditional lethality when exposed to high temperatures (Rendon, 1996).

First-generation transgenic systems sought to improve these technologies by creating analogues of GSSs that could also be used as population suppression measures in the field. The most widely adopted of these was the Release of Insects carrying a Dominant Lethal technology (RIDL) (Thomas et al., 2000), but also see (Schetelig and Handler, 2012) (Ogaugwu et al., 2013; Schetelig et al., 2016). The basis behind RIDL is the genetic modification of a pest to carry a deleterious/lethal gene whose expression can be turned off (repressed) during rearing, but which, when inherited by the progeny of released insects, will result in lethality for individuals in the field. As with SIT, mass releases of RIDL insects can thus suppress a target population by continually killing off field-born individuals before they can reproduce. Female-specific RIDL (fsRIDL) and genetic sexing strains have been developed in many species by combining this repressible lethality with sex-alternatively spliced introns from basal genes within the sex determination pathway (Fu et al., 2007; Schetelig and Handler, 2012; Ogaugwu et al., 2013; Tan et al., 2013), specifically *dsx* and *tra*. The pre-mRNA of these two genes is spliced differently between males and females—leading to the sex-specific inclusion or exclusion of exonic sequences. Sex-specific transgene expression can be designed by including the sequences responsible for sex-specific splicing (introns) embedded within components integral to the repressible-lethal system. As such, functional fsRIDL proteins are produced in one sex (usually females) and not the other (in the same manner as *tra* and sex-specific dsx proteins). Released fsRIDL individuals are homozygous fertile males that produce heterozygous male-only offspring when mating to wildtype females following release. These heterozygous fsRIDL males can then produce wildtype males and females as well as heterozygous fsRIDL males, resulting in a steadily diluting suppressive effect without continued releases. Female-specific RIDL lines have been developed in many insect pest species, including tephritid fruitflies (Fu et al., 2007; Ant et al., 2012), blow flies (Yan and Scott, 2020), screwworms (Concha et al., 2016), moths (Morrison et al., 2012; Jin et al., 2013) and mosquitoes (Phuc et al., 2007; Collado, 2013). Proof-of-principle demonstrations have also been made in beetles (Gregory, 2015). Caged and open-release trials have demonstrated that repeated releases of fsRIDL males can cause the rapid suppression of target populations (Harris et al., 2011; Leftwich et al., 2014; Carvalho et al., 2015; Shelton et al., 2020).

For these first-generation technologies, it is not necessary to know the precise means by which the sex-specific processing of *dsx* or *tra* components are regulated (i.e., the upstream elements of the pyramid which act upon them). All that is required is a basic understanding of the arrangement of the chosen gene and final mRNA differences between sexes. This conservation of function is a distinct advantage for adapting transgenic constructs, with minimal changes, across multiple species (Tan et al., 2013). A further advantage of using highly conserved basal elements of the pyramid is that the splicing signals which regulate their sex-specific splicing are often shared between closely related species (Ant et al., 2012). For example, an fsRIDL construct built using intronic sequences from pink bollworm (*Pectinophora gossypiella*) *dsx* functioned just as effectively in that species as it did in silkworm (*B. mori*) and diamondback moth (*Plutella xylostella*) (Jin et al., 2013; Tan et al., 2013). The limitation of using these downstream elements of the pyramid is that these systems are generally limited to *killing* females rather than their conversion to males. This, coupled with the self-limiting nature of these technologies, makes them far less potent than “second-generation” technologies (next section).

## Second-Generation Technologies: Current State-of-the Art

### Gene Drive

Advances in genome editing, particularly the development of the CRISPR/Cas9 platform, have allowed a new generation of GPM technologies for population suppression to be developed. Commonly referred to as “homing gene drives” (HGD), these second-generation technologies were designed for population suppression or alteration (Burt, 2003; Alphey, 2014) and engineered initially using homing endonuclease genes and, more recently, CRISPR/Cas9. A gene drive is any system in which genes enhance their transmission in a sexually reproducing population above that predicted by Mendelian inheritance (50%). This enhanced transmission is beneficial in a pest control context as it allows “fitness-reducing traits”, e.g., pesticide/environmental susceptibility or sterility, to be spread through a target population by the autonomous action of the gene drive. Homing gene drives encode a nuclease that recognises and cuts a sequence at the target wildtype locus on the homologous chromosome to where the HGD transgene is inserted. Double-stranded DNA breaks are known to be “editogenic,” and under some conditions, cells can use intact homologous DNA as a template to repair the broken DNA. As such, during repair of this break, the broken strands are resected, and the host cell uses the intact HGD-bearing chromosome as a template to fill in the gaps, in a process known as homology-dependent repair (HDR). In using the HGD-bearing chromosome as a template for repair, this sequence copies itself onto the repaired chromosome. If this process happens efficiently and as part of the organism’s germline, most gametes will receive a copy of the HGD (super-Mendelian inheritance). Depending on its imposed fitness costs, the gene drive element may then increase in frequency within a population. With a high transmission efficiency, theoretically extreme enough to spread throughout a population even at a fitness cost to the individual, gene drives have been seized upon by those working on genetic methods for pest control for their ability to engineer populations even at low introduction frequencies (Gantz and Bier, 2015; Gantz et al., 2015; Hammond et al., 2016; Champer et al., 2018) Figure 3. Currently one of the most pressing issues in the “gene-drive” community is the control and safe practice of potential gene drive releases, leading for example, to the development of confinable split-drive technologies that also safeguard against accidental release (Li et al., 2020; López Del Amo et al., 2020).

**FIGURE 3:**
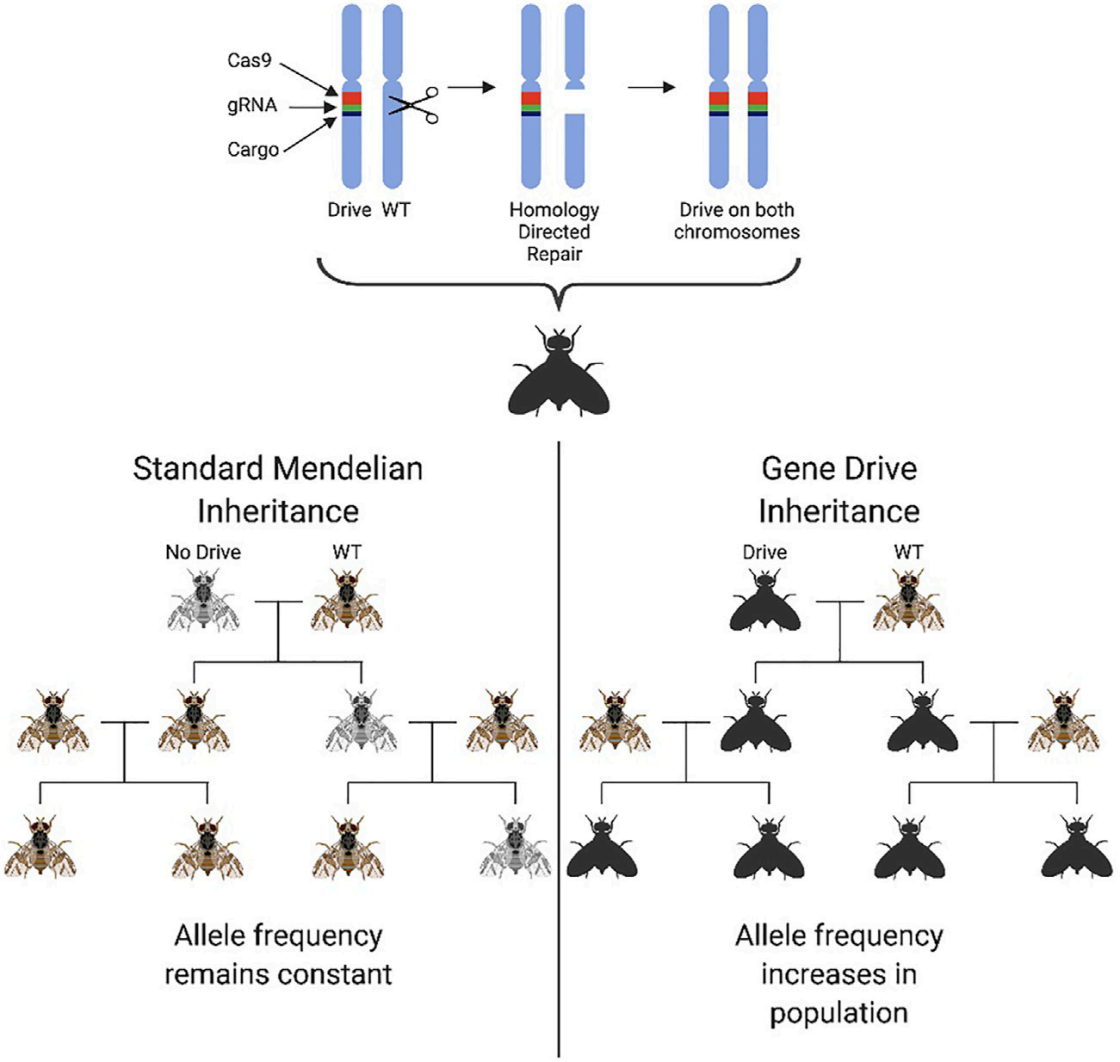
The genetic principle and biased inheritance of a homing gene drive. Homing drives ensure their own transmission to the next generation through homology-directed repair. The Cas9 element of the construct, coupled with specific guides, cut the wildtype chromosome at a precise genomic location triggering a double-strand break. This break is then repaired using homology-directed repair with the remaining chromosome being used as a template. The drive construct is therefore present on both chromosome copies ensuring 100% offspring inheritance. A cargo element is also coupled with the drive elements which can spread a desired trait through a population. This biases the usual 50% inheritance mandated by standard Mendelian genetics and results in the 100% inheritance of the drive in offspring. The drive frequency in the population will therefore increase with subsequent populations as opposed to the standard mendelian inheritance at which the allele frequency would remain at a constant level in the population if a fitness cost is not incurred.

HGDs designed for population suppression are inserted into (and therefore disrupt) a gene of essential function. If that essential gene is haplosufficient, the HGD causes a deleterious recessive phenotype. Heterozygous individuals are viable and contribute to the spread of the drive, but when the drive reaches a high allele frequency in the population, more and more non-viable homozygous individuals are produced, leading to a dramatic reduction in the reproductive capacity of the population (Deredec et al., 2011). The most efficient designs target viability in only one sex (usually females), allowing the drive to spread efficiently within the other sex, regardless of frequency.

As with any pest elimination strategy, selection for resistance to a gene drive is a concern. The most commonly cited mode of resistance is mutations in the target cut sites “resistance alleles,” which prevent further recognition by the Cas9/sgRNA complex and therefore targeting by the drive. If resistance alleles do not severely disrupt the gene’s coding potential (e.g., synonymous mutations, or small in-frame deletions), they may be rapidly selected for in a drive carrying population as the fitness differential between the resistance mutation and the drive is expected to be large (Champer et al., 2017; Carrami et al., 2018). Several strategies can mitigate this, including restricting expression of the gene drive to the germline (Hammond et al., 2021) targeting highly conserved gene sites, hoping that this indicates low tolerance for mutated alleles. In at least one example in *A. gambiae,* this appears to have been achieved by targeting the intron 4 - exon 5 boundary (splice junction) of *dsx* (Kyrou et al., 2018). Typically, this fifth exon is included in female, but not in male, *dsx* mRNA (female-specific exon). However, when the splice junction is disrupted, the 5th exon is instead excluded (skipped) in both sexes. Homozygous deletions at this junction incapacitated female sexual development leading to intersex and sterile females as these individuals could not produce functional dsxF (lacked the 5th exon). Male development and fertility, however, was unaffected as this 5th exon is canonically excluded from the final mRNA transcript in that sex. Within this study, this produced a highly effective drive, spreading female non-viability through a caged population, leading to a rapid population crash (Kyrou et al., 2018).

Much like earlier “first-generation” systems, the use of highly conserved, downstream basal elements of the sex determination pathway as essential components of a suppression drive produced reliable, predictable and effective mechanisms of generating female non-viability (in this case sterility, rather than lethality, as with first-generation technologies). The highly conserved and well-understood role of *dsx* in the downstream regulation of female sexual development means that it is highly likely that similar suppression drives could be developed in a range of other insect pest species. However, there are differences between the number and “style” of exon skipping which occurs between different insect species, which would require consideration.

While no functional resistant alleles were observed in this study, it is possible that at larger-scale releases, pre-existing or *de novo* alleles might eventually occur (Bier, 2021). Including multiple guide RNAs designed against numerous sequences at the target loci, also known as “multiplexing,” is one frequently discussed mitigation against this (Carrami et al., 2018). Pre-existing sequence variations or failed homing attempts must inhibit all target sequences simultaneously to inhibit the drive and are therefore less likely to generate functional resistant mutants (Marshall et al., 2017; Champer et al., 2018, 2020b; Oberhofer et al., 2018; Champer S. E. et al., 2020). However, the small target site of the exon-splice junction in *dsx* means that multiplexing guide RNAs would be difficult to engineer for this gene and would likely be an issue in using homologous targets in other species. Complementary alternatives, including combinate X-shredder drives, have been demonstrated (Simoni et al., 2020).

### Precision-Guided Sterile Insect Technique

One alternative approach to using CRISPR/Cas9 to develop HEGs is to improve “first-generation” technologies with precise gene editing. Coupling the precision of CRISPR/Cas9 gene editing to enhance SIT has been proposed and developed as pgSIT (precision-guided sterile insect technique). This alternative approach to using CRISPR/Cas9 creates sterile males and kills or incapacitates females by targeting both a male fertility gene such as *beta2-tubulin* and elements of the sex determination pathway such as *dsx, tra* or *sxl.* RNA guides targeting *sxl* and *beta2-tubulin* coupled with a *Cas9* under a germline-specific promoter killed female embryos and produced sterile, male-only progeny in *Drosophila.* Targeted knock-out of *dsx and tra* resulted in intersex females (Kandul et al., 2019). Newer developments include a temperature inducible true-breeding strain that eliminates the requirement of maintenance and sexing of two independent parental strains (Cas9 and gRNA) (Kandul et al., 2021) Like suppression gene drives, the downstream, basal elements of the sex determination pathway are a reliable target for female non-viability. Versions of pgSIT have also been developed in mosquitoes (*Ae. aegypti*), and unlike a gene drive, this approach is self-limiting and is not predicted to persist or spread in the environment (Li et al., 2021).

## Third-Generation Technologies: Looking to the Future

Whereas first- and second-generation technologies seek to manipulate or disrupt basal elements of the sex determination pyramid in order to reduce the fitness of or kill females, future “third-generation” technologies may be designed to manipulate the master regulators of sexual fate, to affect full sex conversion. If attached to an efficient gene drive system, such a technology would spread through a target population causing a growing wave of sex distortion. This is theoretically more efficient than a second-generation system that kills or incapacitiates one sex as homozygotes, because all inheritors, regardless of their genotype, continue to spread the system. This increased efficiency could potentially allow for a dramatic reduction in the number and size of releases required for population control. Depending on the efficiency of sex conversion, this could enable threshold-dependent gene drives to be used, previously discounted for population suppression because of their intolerance to high fitness loads (Leftwich et al., 2018; Champer et al., 2020a).

Many aspects of the theory underpinning such third-generation technologies pre-date second-generation strategies (Lyttle, 1991). However, they have proven challenging to enable in practical terms. Part of the reason is the non-conserved nature of the upstream components that need to be manipulated. This requires deep and specific knowledge of master regulators and their web of interactions with downstream elements for each specific pest species to be targeted. Even then, the unpredictable/inflexible nature of “coupled” sex determination and dosage compensation systems may make such a design unachievable in some species. Hence for third generation systems, the transfer of efficient gene drive designs between pests may not always be possible. In a similar vein, if sex-specific components of fitness are sex-linked it may be the case that efficient sex conversion can be achieved, but the sexual competitiveness of converted individuals is diminished.

The mechanisms for enacting sex conversion will vary greatly depending on whether the targeted gene(s) are the master regulators of sex determination (and whether these initiate a male or female cascade), or those genes directly downstream (*tra* or *tra-2*). In an XX/XY system, sex conversion through expression of a master regulator is likely to produce a dominant effect. This is highly likely to affect the dynamics of a gene drive. For example, the effects on population suppression would be seen much earlier than one where conversion is enacted through disruption of a recessive switch e.g., *tra* or *tra-2* (Hoshijima et al., 1991; Pane et al., 2002; Sarno et al., 2010). For systems where the presence of the master regulator determines femaleness [for example, the ZZ/ZW systems common to Lepidoptera (Suzuki et al., 2010)], maleness could be achieved by inactivating the master regulator or making the element below it resistant to its activity [see (Sakai et al., 2016)].

In the next section, we discuss evolutionary and empirical manipulation studies of dosage compensation and sex-linked fitness traits and outline the hurdles these may pose to engineering efficient third-generation technologies.

### Dosage Compensation

In heterogametic sex chromosome systems, the loss of recombination between the dissimilar chromosomes leads to multiple evolutionary processes acting to reduce the size of the sex-limited chromosome, including mutation accumulation and gene loss (Bachtrog, 2013; Bachtrog et al., 2019). This can lead to a monoallelic state for the heterogametic sex, in which a single functional allele is present for multiple genes on the single copy of the X or Z chromosome, and the homogametic sex retains two functional copies. This imbalance of alleles between the sexes is often hypothesised to require dosage compensation mechanisms to restore a balanced state of gene expression: classically, this was thought to occur across the entirety of the X or Z chromosome (Ohno, 1967). If dosage compensation occurs across the entirety of sex chromosomes in a target pest species, it could prove challenging for the design of third-generation technologies, particularly if the dosage compensation pathway is downstream of the master regulator (i.e., the pathways are “coupled”). This is because, while such a system would ectopically express a sex determination master regulator, it would not alter the sex chromosome complement of an individual. If the two pathways are “coupled”, that individual (say, a female) would enact the dosage compensation pathway of the opposite sex (a male), despite having a “female” sex chromosome complement. This would lead to lethal misexpression of sex-linked genes and death, rather than conversion to the opposite sex.

Fortunately for GPM engineers, there is growing evidence from evolutionary studies of an alternative model of gene-by-gene dosage compensation. This alternative model states that only a minority of loci may be dosage-sensitive, specifically genes with particularly high expression levels, or those that have evolved through recent gene duplication. This may have a low correlation with levels of observed sex chromosome divergence (Furman et al., 2020). Where global dosage compensation is primarily observed is in XY systems, and could be driven by the stronger sexual selection and greater reproductive variance in males, this is predicted to result in slower evolution of Z than with X dosage compensation (Mullon et al., 2015). This could mean that sex chromosome dosage compensation may be less of a challenge in ZZ/ZW systems such as Lepidoptera (Gu et al., 2017).

In reality, the nature of dosage compensation appears to vary widely, and exceptions to “general” rules seem to be increasingly common. For example, *A. gambiae* has an XY heterogametic sex-determination system, with a single gene, *Yob,* identified as a Y-linked maleness factor (Krzywinska et al., 2016). The expression of *Yob* begins around 2 hours into embryonic development and precedes that of sex-specific splicing of *dsx* by about 6 hours. Ectopic expression of *Yob* has been confirmed to produce male splice-form *dsx* but leads to female embryonic death while leaving male development unaltered (Krzywinska and Krzywinski, 2018). This pattern of female lethality in the presence of *Yob* can be explained by gene overexpression by both X chromosomes as a result of misapplied dosage compensation leading to female death. Similar experiments in the mediterranean fruit fly (*C.capitata*), which also has an XY heterogametic sex-determination system, have also identified a Y-linked single gene determinant of maleness, *MoY*. Here knockdown of *MoY* was demonstrated to be sufficient to produce total loss of male-specific *tra* mRNA in embryos and complete XY feminisation. Conversely, ectopic expression of *MoY* produced no change in male development and partial or full masculinisation of XX flies (Meccariello et al., 2019). These XX pseudomales were also fertile, demonstrating that there are no genes *essential* to male fertility located on the Y chromosome in medfly. RNAi knockdown of *tra* in several other tephritid fruitflies and *M. domestica* have also produced female to male sex reversal, producing fertile converted males, indicating this approach may be possible in a number of pest insects (Dübendorfer et al., 2002; Pane et al., 2002; Lagos et al., 2007; Concha and Scott, 2009; Hediger et al., 2010; Schetelig et al., 2012) We note though the genetic factors that may influence the outcome of sperm competition have not yet been studied in these systems. In both *An. gambiae* and *C. capitata*; closely related species (*Anopheles arabiensis* with *Yob*; *Bactrocera oleae* with *MoY*), appear to be responsive to their respective male determining signals. However, the fast-evolving nature of the primary sex determination regulators means that these are likely to be restricted to closely related species with either direct homology to these genes or conserved downstream interactions. The disparate responses of female death vs. female-male sex conversion between these two species were entirely unpredictable, and while the role of dosage compensation in this is speculative (and does not preclude that a dosage compensation mechanism exists that is uncoupled from sex determination); evidence to-date indicates these different fates for alteration of sexual development could be the result of just a handful of dosage-sensitive genes on the X chromosome of *An. gambiae* while none are present on the X chromosome of the medfly.

### Essential Male Genes

One prediction of the evolution towards heterogametic sex chromosome systems is the accumulation of sex-specific fitness-enhancing genes on the sex-specific region, often linked to the master regulator through lack of recombination (Mank et al., 2014). As with dosage compensation, this arrangement may prove a hurdle for third-generation sex-conversion systems if these fitness-enhancing genes are not included alongside the master regulator. The Y chromosome of *D. melanogaster* contains male fertility factors. However, it contains only 16 protein-coding genes, and not all are essential for male fertility (Kaiser and Bachtrog, 2010; Zhang et al., 2020). Other examples of essential genes in males can be seen with the engineered manipulation of the male master regulator in *Ae. aegypti* and *Ae. albopictus*. These two mosquitos do not possess hetermorphic sex chromosomes, only a small, non-recombining male-determining region (M-locus) on the short arm of chromosome 1, an otherwise homomorphic autosome (Hall et al., 2014). A single gene *Nix* [a putative recent duplication of *tra2* (Gomulski et al., 2018)], has been identified as the master male determining gene in these species (Liu et al., 2019; Aryan et al., 2020). In *Ae. aegypti*, stable transgenic expression of *Nix* was sufficient to produce sex conversion of genotypic females into males. Dosage compensation in a species without a heterogametic sex would be unlikely, and the observed sex conversion over female death was in line with this prediction. However, while *Nix* was sufficient for determining male sexual fate, the resultant pseudomales were incapable of flight as they lacked another gene *myo-sex*, also present in the M-locus, which is required for proper development of flight muscles in males (Aryan et al., 2020). Conversely, when analogous experiments were conducted in *Ae. albopictus* it was found that converted pseudomales were not only viable but capable of flight (Lutrat et al., 2022). Interestingly, despite evidence of an M-linked *myo-sex* gene, converted pseudomales could still express comparable levels of *myo-sex* transcripts to wildtype males. These results indicated at least one duplicate copy of *myo-sex* exists which is not M-linked in this species. These converted pseudomales, displayed reduced competitiveness compared to wildtype males, suggesting the possibility that the M-locus in this species may harbour other, as yet unknown, male fitness-enhancing genes. However, this is difficult to disentangle from the adverse effects of transgenesis (including ubiquitous marker gene expression, disruption of essential genes at the insertion sites or incomplete masculinization). These findings highlight that, even in species without apparent dosage compensation or heteromorphic sex chromosomes, efficient sex conversion technologies may prove more challenging to engineer than simply transgenically expressing the master regulator. Additionally, the significant differences between these two closely related species suggest that substantial fundamental research will be required to underpin the development of these technologies in novel pests.

## Conclusion

Manipulating sex determination pathways for genetic pest management has many potential applications. Previous technologies have used the highly conserved “basal” elements of *dsx* and *tra* common to almost all insect species to produce reliable mechanisms of biasing sex ratios with the release of modified males carrying factors to generate female sterility or death. Newer technological developments, including homing gene drives, demonstrate these basal elements continue to be predictable and reliable targets for control. Looking forward, development of genetic editing techniques to manipulate “master regulators” of sexual fate have the potential to improve the performance of a wide variety of genetic control methods. However, this approach has potential challenges—different species may exhibit sex-linked genes that are vital for viability or sexual fertility or have strong dosage compensation. However, this is a vibrant field of research and much experimental work is ongoing in a range of different pest species. While it is likely that the application of sex conversion for pest control will inevitably be applied on a case-by-case basis, active investigations on a number of fronts are likely to improve our understanding of the basic biology and evolution of sex determination, as well as expand our genetic toolbox for applied pest management.

BOX 1 **Alternative methods to altering sex ratios outside of the sex determination pathway**

Two modifications are principally required for Genetic Sexing Strains (GSS) 1) introduction of a recessive conditional lethal gene or viable selectable recessive colour mutations and 2) translocation of a wild-type rescue allele onto the male Y-chromosome. In the final strains, females are homozygous for one or more selectable mutations, while males are heterozygous with a wildtype phenotype (Rendon, 1996). These strains are highly effective at producing substantial bias in the reproductive sex ratios or enabling efficient sex separation, but because of the mutations and chromosome translocations required to generate these strains, high levels of sterility and rearing difficulties were common, many strains were also unstable as a result of these complex chromosomal rearrangements (Robinson, 2002; Nguyen et al., 2021).

Genetic sexing strains produced strong genetic male bias, achieved not through manipulation of the sex determination pathway, but by positioning autosomally lethal alleles onto the sex chromosomes. The advent of powerful genome editing tools and synthetic biology has allowed for the development of other, more refined artificial sex distortions such as X-shredding. These systems exploit the heterogametic nature of these species, where fathers always transmit their X chromosome to their daughters and the Y chromosome to their sons, to cause lethal changes to essential genes, without involving the sex determination pathways directly.

X-shredders were first pioneered in *Anopheles gambiae* when I-PpoI was discovered to cut the X chromosome in several locations due to its targeting of the repeated ribosomal rDNA (Windbichler et al., 2007). By engineering a destabilised version I-PpoI its activity could be restricted to male meiosis thereby ensuring males were unable to pass on a functional X chromosome (Galizi et al., 2014). As opposed to using an endonuclease which targets a conserved repetitive element; X-shredding can also be driven using CRISPR/Cas9 and targeted sgRNAs, with Cas9 cleavage limited to the male germline (Galizi et al., 2016; Fasulo et al., 2020; Meccariello et al., 2021).

Y-linked editors have also been proposed as a self-limiting strategy significantly more effective than those previously proposed (Burt and Deredec, 2018). If released simultaneously with an autosomal X-shredder this efficiency can be further increased. An alternative approach would be to drive an X-shredder from the Y-chromosome to ensure male offspring inheritance (Gamez et al., 2021). Other proposed Y-linked suppression systems include Medusa; combining a maternally-expressed, X-linked toxin and a zygotically-expressed, Y-linked antidote that causes suppression of the female population and selects for the transgene-bearing Y. At the same time, a zygotically-expressed, Y-linked toxin and a zygotically-expressed, X-linked antidote selects for the transgene-bearing X in the presence of the transgene-bearing Y to create a threshold dependent, highly male-biasing suppression system (Marshall and Hay, 2014) present its own challenges as expression during spermatogenesis can be difficult to achieve from the Y chromosome due to transcriptional repression (Alcalay et al., 2021).
